# A Preliminary Approach to Oral Low-Dose Ketamine Self-Administration in Mice (*Mus musculus*)

**DOI:** 10.3390/cimb47080592

**Published:** 2025-07-27

**Authors:** Cláudia A. Rocha, Luís Sampaio, Luís M. Félix, Sandra M. Monteiro, Luís Antunes, Carlos Venâncio

**Affiliations:** 1 School of Life and Environmental Sciences (ECVA), University of Trás-Os-Montes and Alto Douro (UTAD), 5000-801 Vila Real, Portugal; xana3@live.com.pt (C.A.R.); luis.carlos.sampaio98@gmail.com (L.S.); smonteir@utad.pt (S.M.M.); 2 Centre for the Research and Technology of Agro-Environmental and Biological Sciences (CITAB), University of Trás-Os-Montes and Alto Douro (UTAD), 5000-801 Vila Real, Portugal; lantunes@utad.pt (L.A.); cvenanci@utad.pt (C.V.); 3 Institute for Innovation, Capacity Building and Sustainability of Agri-Food Production (Inov4Agro), University of Trás-Os-Montes and Alto Douro (UTAD), 5000-801 Vila Real, Portugal; 4 Department of Animal Science, School of Agrarian and Veterinary Sciences (ECAV), University of Trás-Os-Montes and Alto Douro (UTAD), 5000-801 Vila Real, Portugal; 5 Veterinary and Animal Research Centre (CECAV), Associate Laboratory of Animal and Veterinary Sciences (AL4AnimalS), University of Trás-Os-Montes and Alto Douro (UTAD), 5000-801 Vila Real, Portugal

**Keywords:** ketamine, low doses, mice, oral administration, oxidative stress

## Abstract

With ketamine gaining attention as a therapeutic drug, oral administration offers an effective alternative to traditional parenteral routes. However, a significant gap remains in understanding its use via voluntary ingestion. This preliminary study aimed to explore the feasibility of oral ketamine self-administration in mice (*Mus musculus*), while investigating the effects of low concentrations on the brain, liver, and kidney. Adult mice were divided into three groups and received ketamine in their drinking water for 16 days at 0 (control), 5 (K5), or 10 mg/L (K10). A transient decrease in water consumption was observed in both sexes in the K10 group; however, only females in this group showed differences in ketamine intake between groups on some days. Oxidative stress markers measured in the brain, liver, and kidney only revealed higher catalase activity in the brains of females. No significant alterations were observed in liver and kidney function in either sex, nor in inflammation, apoptosis, or DNA damage in kidney tissues. Overall, these findings support the viability of voluntary oral ketamine administration and accentuate the need to refine the proposed model, not only to prevent water consumption inhibition but also to extend the exposure period, explore potential sex-related differences in ketamine intake, and further confirm the safety of oral ketamine administration.

## 1. Introduction

Ketamine is a commonly used anaesthetic with a very complex pharmacology, since it acts as a non-competitive antagonist of the N-methyl-D-aspartate (NMDA) receptors, but it can also exhibit effects through glutamate-independent mechanisms [1]. As a result, ketamine is associated with a range of adverse effects, which limit its use to few specific situations [2,3]. Nevertheless, interest in ketamine as a therapeutic drug has grown significantly over the years as multiple studies have reported ketamine’s analgesic and antidepressant properties at lower doses [4,5,6,7,8,9,10,11,12,13,14,15,16]. The antidepressant effects of these lower doses are typically documented in the two hours after the drug’s administration and remain effective for up to a week [17,18]. Similarly, lower doses of ketamine have proved to be effective in acute, chronic, and neuropathic pain management, with its analgesic effects lasting for at least 24 h with minimal adverse effects [19]. These findings indicate that ketamine exerts both rapid and persistent responses and suggests that its metabolites may also play a part in the duration of effects [20,21,22]. Due to its robust response, an urgent need to expand the knowledge on less invasive methods for administering ketamine, such as the oral route, has also emerged [15,16].

Despite its attractive properties, long-term administration of ketamine is often associated with an increased oxidative stress environment, especially in organs that are particularly susceptible to oxidative damage, such as the brain, liver, and kidney, with evidence revealing increased reactive oxygen species (ROS) levels, lipid peroxidation, and altered antioxidant enzyme activity [23,24,25,26,27]. The kidney has an increased potential for drug-induced damage due to its direct role in filtering and excreting chemical agents, which can translate into more severe consequences [28,29]. Notably, these effects are primarily described in association with high doses of ketamine administered through intraperitoneal, subcutaneous, or intravenous routes [23,30,31]. On the other hand, the effects of lower ketamine doses administered orally remains poorly understood. In animal studies, oral ketamine is typically administered via oral gavage, a method that allows for precise dosing but can cause physical stress and confounding physiological effects, such as increased blood pressure, heart rate, and plasma corticosterone levels, which might affect the animal’s welfare and interfere with experimental results [9,31,32,33,34]. To address these limitations, voluntary oral administration of target drugs has been explored as a less stressful alternative [34,35]. However, studies on oral low doses tend to focus on ketamine’s therapeutic or acute adverse effects, leaving a significant gap concerning their long-term impact [15,16].

While rodent models for gavage and parenteral administration of ketamine and its effects are well documented, voluntary oral self-administration remains largely unexplored, delaying the implementation of less invasive drug delivery models in different settings. Thus, the objective of this study was to establish a preliminary murine model of oral ketamine self-administration and investigate the potential adverse effects of low concentrations on the brain, liver, and kidney.

## 2. Materials and Methods

### 2.1. Animal Housing and Maintenance

Thirty-eight FVB/n mice (*Mus musculus*), bred in the animal facility of University of Trás-os-Montes and Alto Douro (UTAD), were paired in polycarbonate 1284L Eurostandard Type II L cages (Tecniplast, Milan, Italy) based on age (5–9 months) and sex (20 females and 18 males). The mice (27.4 g ± 4.6 g) were housed in a room in the same facility with a controlled 12:12 h light–dark cycle, temperature (23 °C ± 2), and humidity (50% ± 10) [36]. The animals were provided with 30 mL of tap water, a standard diet (4RF1; Mucedola, Milan, Italy) ad libitum, and a standard corncob litter (Corncob ultra 12, Ultragene, Viseu, Portugal) as bedding material along with cotton for nesting. The animals were acclimatized to these conditions for two weeks.

### 2.2. Experimental Design

**Figure 1:**
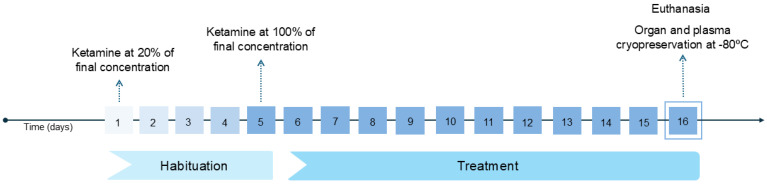
A timeline of the procedure from the first ketamine administration to the organ collection and cryopreservation. Ketamine’s concentration gradually increased during the habituation period from 20% (day 1) of its final concentration to 100% (day 5). During the treatment period (days 5–16), the administered ketamine concentration (K5: 5 mg/mL; K10: 10 mg/mL) was not altered.

The mice were randomly divided into three groups: the control, receiving 0 mg/mL (*n* = 4/sex), and two treatment groups receiving 5 (K5; *n* = 8/sex) or 10 mg/mL (K10; *n* = 6 males, 8 females) of ketamine (100 mg/mL; Nimatek; Dechra, Lisbon, Portugal) diluted daily in their drinking water for a period of 16 days [11,14,37]. Ketamine was introduced into the water at 20% of the final concentration (K5: 1 mg/mL; K10: 2 mg/mL) to allow the mice to acclimate to the solution and reduce the possibility of aversion associated with the altered water (Figure 1). Over the first five days, concentration gradually increased until the final concentrations of 5 (K5) and 10 (K10) mg/mL were reached. At the end of the study, the mice were euthanized by overdose, using an intraperitoneal injection of sodium pentobarbital (145 mg/kg; Euthasol^®^ 400 mg/mL, Esteve, Barcelona, Spain). Post-euthanasia, blood samples were collected into lithium-heparin tubes and centrifuged for 15 min at 4 °C and 1400× *g*. The resulting plasma was transferred to clean tubes and stored at −80 °C alongside the brain, liver, and right kidney from each mouse. All sample processing and analysis were performed blind to each mouse treatment group.

### 2.3. Water Consumption and Ketamine Intake

The consumption of water (mL), in which ketamine was diluted, was measured daily by recording the volume consumed from each cage’s water tube. This measure was registered daily to monitor ketamine intake and ensure that ketamine administration did not impact on the animals’ normal activities. The amount of ketamine daily ingested per cage was then calculated using the daily concentration administered in the water (mg/mL), water intake (mL), and average weight (kg) of the female or male mice in each cage following Equation (1). The average ketamine doses ingested per group were also calculated.

(1) $$Ketamine\;intake\;\left(mg/kg\right)=\frac{ketamine\;dose\;\left(mg/mL\right)\times water\;intake\;\left(mL\right)}{average\;weight\left(kg\right)}$$

### 2.4. Oxidative Stress Quantification in Brain, Liver, and Kidney Samples: Sample Preparation and PROTEIN Quantification

The whole brain and a random piece of liver (0.6 g) and kidney (0.05 g) were placed in separate microcentrifuge tubes and prepared for the analysis of different oxidative stress parameters [38]. ROS levels were quantified using dichlorofluorescein (DCF) standards (0–1 mM), malondialdehyde (MDA) levels using MDA standards (0–100 mM), and protein carbonyl (PC) levels assuming the absorption coefficient of 22.0 mM/cm [38,39,40]. Superoxide dismutase (SOD; 0–150 U/mL), catalase (CAT; liver: 0–480 U/mL; brain and kidney: 0–60 U/mL), glutathione reductase (GR), glutathione peroxidase (GPx), and glutathione s-transferase (GST) levels were quantified using their respective standards following established protocols [41,42,43,44].

For the reduced (GSH) and oxidized (GSSG) glutathione tests, the samples were prepared following a different method, and the results were quantified using GSH (0–1000 mM) and GSSG (0–1000 mM) standards [45,46,47].

The total amount of protein in each sample was quantified at 280 nm, using a power wave XS2 microplate scanning spectrophotometer (Bio-Tek Instruments, Winooski, VT, USA).

### 2.5. Analysis of Serum Biomarkers to Evaluate Liver and Kidney Function: Alanine Aminotransferase (ALT), Aspartate Aminotransferase (AST,) and Alkaline Phosphatase (ALP) Levels as Indicators of Liver Function, and Creatinine and Serum Urea Nitrogen (BUN) Levels as Indicators of Kidney Function

ALT (ref. number: 41282), AST (ref. number: 41272), ALP (ref. number: 41242), creatinine (ref. number: 1001111), and BUN (ref. number: 1001333) levels were estimated according to the protocol described in the kit by Spinreact (Girona, Spain).

### 2.6. Evaluation of Additional Parameters in Kidney Samples

Given the kidney’s increased susceptibility to drug-induced damage [29], additional assessments were conducted to evaluate apoptosis (caspase 3 and caspase 9), inflammation (nitric oxide [NO]), and nucleic acid damage (DNA double-strand breaks estimation).

#### 2.6.1. Caspase 3 and 9 Levels as Apoptosis INDUCTION Markers

The activity of both caspase 3, the primary effector of apoptosis, and caspase 9, activated through the intrinsic pathway, served as apoptosis markers in the kidney [48,49]. Samples were incubated with a caspase buffer, and their respective substrates, following a standard protocol [50]. The results were quantified using *p*-nitroaniline (pNA) standards (0–500 mM) [50].

#### 2.6.2. NO as an Inflammation Marker

Nitric oxide (NO) levels were estimated using Griess reagent and 0.1% N-(1-Naphthyl) ethylenediamine dihydrochloride in a 1:1 proportion and quantified using sodium nitrate (NaNO_2_) standards (0–0.1 mM) [51].

#### 2.6.3. DNA Double-Strand Break Estimation as a DNA Damage Marker

Double-strand breaks were estimated using 2% SDS buffer followed by 0.12 M potassium chloride (KCl) and incubation at 60 °C [52]. Samples were centrifuged, and the supernatant was incubated with Hoechst 33258 dye diluted in buffer [52]. The results were quantified using DNA standards diluted in TAE 1× [52].

### 2.7. Data Analysis

Statistical analyses were performed using GraphPad Prism 9 for Windows (Version 9.5.0; La Jolla, CA, USA). The normal distribution and homogeneity of the data were evaluated by the Shapiro–Wilk and Brown–Forsythe tests, respectively. Water and ketamine intake were analyzed by two-way analysis of variance (ANOVA) followed by Šidák’s multiple comparison test, with results presented as the mean and standard deviation (SD). For the remaining parameters, normally distributed data was analyzed by a one-way ANOVA followed by Tuckey’s pairwise comparison test and the results are presented as the mean and SD. The data that did not follow a normal distribution was evaluated using the Kruskal–Wallis non-parametric test followed by Dunn’s pairwise comparison test, and the results are presented as the median and interquartile range (IQR). For all analyses, a significance level of 5% (*p* < 0.05) was established. Each analysis was conducted with at least 3 replicas from each group.

## 3. Results

### 3.1. Water and Ketamine Intake

**Figure 2:**
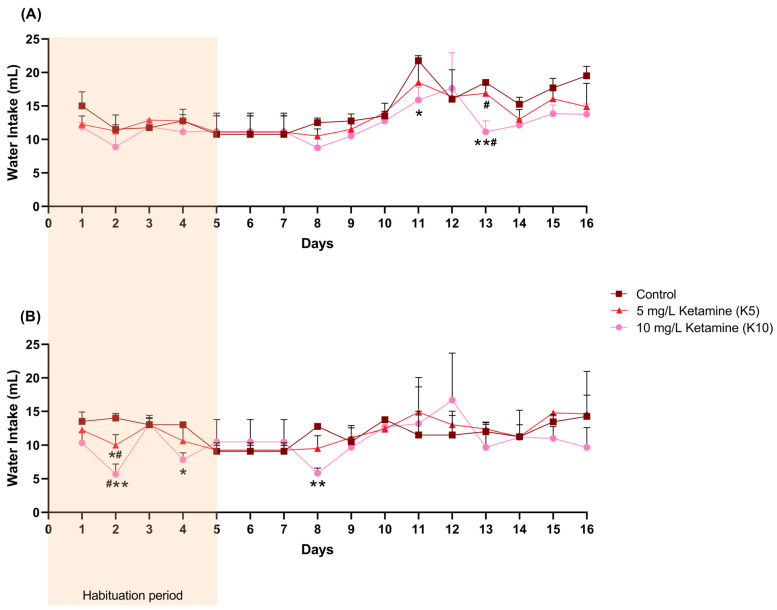
The female (**A**) and male (**B**) daily water intake (mL) per cage (control: 2 cages; K5: 4 cages; K10: 4 cages), including the initial habituation period of 5 days during which ketamine concentration was gradually increased. Statistical analysis was performed using ANOVA followed by Šidák’s multiple comparison test for each day, and the results are presented as mean and SD. * indicates significant differences from the control group (* *p* < 0.05, ** *p* < 0.01), and # indicates significant differences between the K5 and K10 groups (# *p* < 0.05).

Female water intake (mL) varied significantly over time (F (3.689, 25.82) = 14.50, *p* < 0.0001), with a significant reduction observed in the K10 group compared to the control group on days 11 (95% confidence interval (CI) = 1.993, 9.757, *p* = 0.0147) and 13 (95% CI = 3.923, 10.83, *p* = 0.0061), and compared to the K5 group on day 13 (95% CI = 1.824, 9.676, *p* = 0.01) (Figure 2A, Table S1).

In male mice, water intake (mL) in the K10 group was only significantly lower than in the control group on days 2 (95% CI = 3.994,12.67, *p* = 0.0087), 4 (95% CI = 1.627, 8.707, *p* = 0.0241), and 8 (95% CI = 4.747, 9.086, *p*= 0.0021), and lower than the K5 group on day 2 (95% CI = 0.3391, 8.328, *p* = 0.0379) (Figure 2B, Table S2). In addition, the K5 group also revealed a lower consumption when compared to the control group on day 2 (95% CI = 0.6557, 7.344, *p* = 0.0282).

**Figure 3:**
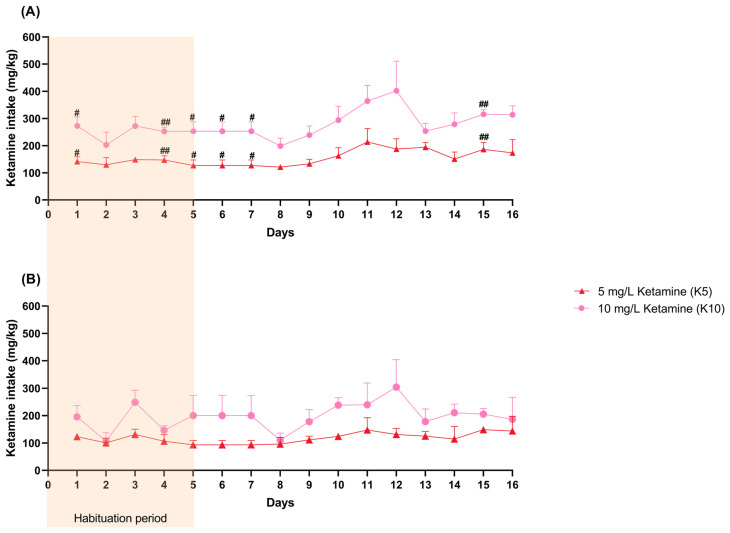
The female (**A**) and male (**B**) daily ketamine intake (mg/kg) per cage (control: 2 cages; K5: 4 cages; K10: 4 cages) calculated based on daily water consumption (mL) and the average weight (kg) of the mice. The habituation period is visually represented in the figure (days 1–5). Statistical analysis was performed using ANOVA followed by Šidák’s multiple comparison test for each day, and the results are presented as mean and SD. # indicates significant differences between the K5 and K10 groups (# *p* < 0.05, ## *p* < 0.01).

Female ketamine intake (mg/kg) varied significantly over time (F (3.252, 19.51) = 8.9946, *p* = 0.0005) and with the administered dose (F (1, 6) = 287.6, *p* < 0.0001), with a significant interaction between time and dose (F (15, 90) = 1.859, *p* = 0.038), indicating that intake patterns differed between treatment groups throughout the study. Female mice from the K10 group revealed a significantly higher ketamine intake on days 1 (95% CI = −238.0, −23.68, *p* = 0.022), 4 (95% CI = −157.7, −52.42, *p* = 0.0013), 5 (95% CI: −239.0, −18.77, *p* = 0.0251), 6 (95% CI: −233.9, −18.77, *p* = 0.0251), 7 (95% CI: −233.9, −18.77, *p* = 0.0251), and 15 (95% CI: −207.8, −50.89, *p* = 0.0047) (Figure 3A, Table S3).

Male ketamine intake yielded similar results as it varied significantly over time (F (2.156, 10.78) = 4.051, *p* = 0.0465) and with the administered dose (F (1, 5) = 74.22, *p* = 0.0003,), with a significant interaction between the two (F (15, 75) = 1.971, *p* = 0.0289). Nevertheless, no differences between groups were observed on specific days (Figure 3B, Table S4).

Although the precise ketamine intake for each mouse could not be determined, an average estimation per group was calculated. Female mice in the K5 group ingested an average of 154.2 ± 12.5 mg/kg, while the K10 group consumed an average of 275.9 ± 7.1 mg/kg. Male mice from the K5 group had an average ketamine intake of 117.9 ± 12.5 mg/kg, while the K10 group consumed an average of 196.9 ± 11.2 mg/kg. Despite receiving the same concentrations, females and males differed significantly in average ketamine intake within both K5 (*p* = 0.0062) and K10 (*p* < 0.0001).

### 3.2. Oxidative Stress Evaluation

CAT levels were significantly higher than the control in the brains of female K5 (*p* = 0.0156) and K10 (*p* = 0.032) group mice (Table 1), but no significant changes were observed in the brains of male mice. The oxidative stress parameters were not significantly altered in the liver (Table 2) and kidney (Table 3) of the female or male mice.

### 3.3. Liver and Kidney Function Assessment

Ketamine demonstrated no significant effect on the liver of the treated mice, as indicated by the results for AST, ALT and ALP activity. Similar results for kidney function were observed, as ketamine was revealed to cause no significant changes in creatinine and BUN levels (Table 4).

### 3.4. Apoptosis, Inflammation, and DNA Damage in Kidney Tissues

The apoptosis, inflammation, and DNA damage results showed no significant damage in the kidney tissues or serum of the tested mice (Table 5).

## 4. Discussion

Ketamine has recently become a valuable pharmacological research target due to its exceptional properties, especially when administered in lower doses through less invasive methods, such as the oral route [5,53]. However, as most animal research focuses on higher doses of ketamine and different administration routes, there is limited knowledge on the effects of oral self-administration at lower doses [30]. This study is the first to address this gap by establishing a murine model of oral ketamine self-administration, while assessing the effects of lower and potential therapeutic concentrations on the brain, liver, and kidney. The increase in ketamine concentration transiently decreases water consumption in both sexes; however, the amount of ketamine ingested is only different between groups in female mice. Moreover, despite altering CAT activity in the brains of female mice, ketamine did not affect other oxidative stress parameters in the brains of males, or in the kidney and liver of either male or female mice, suggesting that the oxidant environment might not have been altered. Accordingly, these concentrations did not impact liver or kidney function, nor did they influence apoptosis, inflammation, or DNA damage in kidney tissues.

When administrated orally, ketamine undergoes a significant first-pass metabolism and is converted into norketamine, a major metabolite thought to contribute to ketamine’s therapeutic effects [19,22,54,55]. This metabolic process shortens the drug’s half-life during the elimination phase, promoting rapid elimination and reducing exposure to ketamine, which might help minimize adverse effects [19,54,55]. Despite its low bioavailability when administered orally (16–30%), several studies have documented its rapid therapeutic efficacy [18,19,20,31]. In this study, ketamine was diluted in the drinking water provided ad libitum, allowing mice to consume it freely. To ensure their welfare, mice were housed in pairs, which introduced a limitation to the study as water and ketamine intake could not be accurately controlled and individual variability could be present. Nevertheless, based on the average water consumption per cage, intake remained within the normal range for the species, with only occasional differences between treatment and control groups that might indicate that ketamine had a slight impact on overall water consumption [56]. In addition, mice in the highest concentration group reported the lowest water intake, which may be related to changes in water taste due to the higher drug levels, as ketamine is known to have a bitter taste [57]. Interestingly, daily ketamine intake only differed between groups on some days in female mice, evidencing a different intake profile between sexes and suggesting gender-related sensitivity. This observation aligns with the existing literature but could be further explored when it comes to oral ketamine [58,59,60,61]. The distributed consumption of ketamine throughout the day, combined with its pharmacokinetic characteristics after oral administration, possibly facilitated its gradual elimination, further minimizing the duration of drug exposure and explaining the reduced adverse effects observed in the brain, liver, and kidney [62,63].

Organs like the brain, liver, and kidneys, are particularly susceptible to oxidative damage and related diseases [64,65,66]. This vulnerability arises from the brain’s high oxygen demand, the liver’s intense metabolic activity, and the kidney’s role in filtering and excreting toxins and chemical agents [65,67,68,69]. The primary cause of oxidative damage is ROS, which are naturally present in the body at low concentrations [70,71]. Antioxidant systems, including antioxidant enzymes and non-enzymatic mechanisms, regulate ROS to prevent toxicity [72,73,74]. However, excessive ROS levels can overwhelm these defense systems, leading to oxidation of lipids, carbohydrates, proteins, and nucleic acids with the potential to trigger other biological processes such as apoptosis, necrosis, and inflammation [74]. In this study, while the brain revealed significant changes in an isolated oxidative marker, the liver and kidney reported no alterations, which might suggest that the overall antioxidant capacity may not have been affected. Conversely, the toxicological effects of ketamine administered via conventional routes are well documented in rodents, with several reports revealing increased oxidative stress in the tested organs [24,26,53,75,76]. Still, contradictory findings also exist, as some studies have reported that ketamine administrated subcutaneously in rats or per gavage in mice does not induce oxidative damage in the liver [14,77], while others demonstrate antioxidant effects in rats with traumatic brain injury or acute spinal cord injuries [78,79]. These inconsistencies accentuate the intricacy of ketamine’s pharmacology, which can have a varying impact on different organs, reinforcing the need to fully understand its oxidative impact.

The kidney has an increased potential for drug-induced damage and, as such, a more thorough investigation of the effects of ketamine on this organ was conducted [29]. The kidney is responsible for filtering waste products, including drugs and their metabolites, from the blood and excreting them through urine [29,69,80]. Thus, when kidney function is compromised, this filtration process becomes less efficient, resulting in increased residues in the serum [80,81]. Creatinine and urea, nitrogenous end products of metabolism, are largely eliminated in urine and thus extensively used as biomarkers for assessing kidney function [81]. The results demonstrated no significant alterations in creatinine and BUN levels, which indicates that kidney function may not have been affected by ketamine or its metabolites. Furthermore, the average creatinine and BUN levels were within the range observed in identical strains, further confirming the absence of renal complications [82,83]. A previous study demonstrated renal complications at 300 mg/kg administered daily by oral gavage [31], a cumulative dose similar to the ones reached in this study through gradual self-ingestion over multiple days, yet without evident complications. Nevertheless, another study in rats receiving 15 mg/kg of ketamine by oral gavage for 28 days [84] revealed no signs of toxicity, indicating that this route can still be viable if much lower doses are used. Collectively, the findings available on oral administration reinforce the complexity of ketamine’s action, suggesting that both the dose and method of administration may influence its toxicity profile. In alignment with findings on oxidative stress in the kidney, no significant alterations in the additional parameters were observed, which suggests that ketamine might have a specific range of therapeutic doses that do not significantly impact kidney function, oxidative stress, inflammation, apoptosis, or DNA integrity. In addition, these findings accentuate the discrepancies between the effects observed with higher doses administered through conventional methods and the lower doses administered orally, which warrants further investigation. Nevertheless, the number of studies reporting the consequences of oral ketamine administration—particularly via voluntary administration—is still limited, which leaves a significant gap in the field that the present study begins to fill.

Despite the lessons learned, this study faces some limitations that should be addressed in future research. A primary limitation is the inability to precisely monitor individual water consumption, as water was available ad libitum and measured per cage. This makes it difficult to determine each mouse’s exact ketamine intake and represents a major vulnerability of this method compared to conventional routes where precise dosing is possible. However, the advantages associated with voluntary oral administration are still significant. As a less invasive method, this approach aligns closely with animal welfare principles due to minimal handling procedures and associated stress, which consequently reduces confounding variables. In this light, this study yielded interesting results that could serve as a foundation for future studies aiming to refine the oral ketamine self-administration model, potentially combining a flavoring agent to mask ketamine’s taste and extending the exposure period to fully understand how its absorption, distribution, metabolism, and excretion shape its biological impact. Moreover, including pharmacokinetic analyses that verify whether therapeutic plasma levels are reached, as well as expanded testing of other relevant parameters, would also be a valuable addition to complement these results. Finally, the differences in ketamine intake profiles between sexes warrant further investigation to better understand sex-specific responses and ultimately confirm the safety of oral ketamine administration.

## 5. Conclusions

Ketamine at lower doses is emerging as a promising treatment for depression and pain conditions, with several studies documenting the rapid improvement of these symptoms following oral administration. While some limitations were addressed, this preliminary approach demonstrates the feasibility of voluntary oral ketamine intake in mice by revealing that mice consume ketamine-dosed water. In addition, these findings demonstrate the potential of sex-based differences in intake that may have an influence on the observed outcomes—an aspect warranting further investigation. The results presented could provide a solid basis for future studies aimed at improving this murine model of voluntary oral ketamine self-administration, specifically by including new strategies that enhance the accuracy of dose monitoring and palatability, as well as exploring extended exposure periods and different test parameters. Refining this non-invasive approach could lead the way for broader research into the therapeutic potential of oral ketamine that ultimately confirms the safety and efficacy of this administration route.

## Figures and Tables

**Table 1:** Evaluation of oxidative stress parameters on the brain after exposure to 5 (K5) or 10 (K10) mg/L of ketamine. The experimental groups have at least four samples (*n* = 4) each ^1^.

	Female			Male		
	Control	K5	K10	Control	K5	K10
**ROS**	24.31 (23.75–28.71)	23.42 (20.66–27.18)	27.55 (25.10–29.05)	27.58 (25.62–29.42)	27.17 (24.91–28.57)	27.73 (24.86–32.07)
**MDA**	2.20 ± 0.27	2.03 ± 0.35	2.36 ± 0.50	2.24 ± 0.90	2.05 ± 0.55	2.36 ± 0.84
**PC**	7.63 (7.28–8.41)	7.27 (7.05–7.29)	7.26 (6.25–7.28)	7.276 (7.25–7.30)	7.277 (7.22–7.41)	7.275 (7.19–7.30)
**GSH**	0.15 ± 0.13	0.088 ± 0.08	0.091 ± 0.07	0.14 ± 0.05	0.13 ± 0.08	0.07 ± 0.08
**GSSG**	9.72 ± 4.40	13.82 ± 3.36	11.89 ± 3.05	12.00 ± 3.07	12.57 ± 3.17	13.54 ± 2.91
**SOD**	31.10 (29.25–32.81)	35.12 (28.16–33.93)	35.40 (30.69–40.83)	36.87 ± 0.93	39.14 ± 4.76	41.54 ± 7.62
**CAT**	5.38 (5.19–6.23)	8.30 (7.82–9.46) *	8.32 (6.86–9.78) *	8.85 ± 1.52	7.23 ± 1.74	10.70 ± 4.74
**GR**	8.49 ± 1.51	13.69 ± 5.59	12.28 ± 5.55	14.30 ± 1.84	9.11 ± 2.68	13.66 ± 11.53
**GPx**	20.15 ± 2.87	18.77 ± 6.36	21.82 ± 11.02	26.55 ± 5.22	27.83 ± 5.57	22.95 ± 8.74
**GST**	39.94 (38.55–51.77)	53.16 (48.27–56.43)	52.99 (45.48–71.52)	58.89 ± 13.21	57.35 ± 9.59	69.10 ± 8.59

^1^ Statistical analysis for non-parametric data was performed using the Kruskal–Wallis non-parametric test followed by Dunn’s pairwise multiple comparison test, and data is presented as the median and IQR (25th–75th quartile). For parametric data, ANOVA followed by Tuckey’s pairwise comparison test was used, with results presented as mean ± SD. * indicates significant differences from the control groups (* *p* < 0.05). ROS: µmol DCF/mg protein; MDA: µmol MDA/mg protein; PC: nmol NADH/min·mg protein; GSH: µmol GSH/mg protein; GSSG: µmol GSSG/mg protein; SOD: U/mg protein; CAT: U/mg protein; GR: nmol NADPH/min·mg protein; GPx: nmol NADPH/min·mg protein; GST: nmol CDNB/min·mg protein.

**Table 2:** Evaluation of oxidative stress parameters on the liver after exposure to 5 (K5) or 10 (K10) mg/L of ketamine. The experimental groups have at least four samples (*n* = 4) each ^1^.

	Female			Male		
	Control	K5	K10	Control	K5	K10
**ROS**	12.73 ± 2.92	12.05 ± 7.97	18.90 ± 5.05	19.10 ± 2.40	14.48 ± 6.36	17.34 ± 2.931
**MDA**	3.60 (2.06–3.69)	1.93 (1.03–2.48)	2.14 (1.48–2.78)	2.77 ± 0.44	2.11 ± 1.14	1.78 ± 0.81
**PC**	1.82 ± 0.18	1.70 ± 0.57	1.37 ± 0.41	1.23 ± 0.62	1.61 ± 0.54	1.47 ± 0.50
**GSH**	348.4 (309.8–381.0)	414.7 (317.4–524.1)	393.0 (305.6–522.4)	239.0 ± 129.4	449.5 ± 263.3	581.0 ± 194.6
**GSSG**	1511 (1371–1531)	1835 (1583–2173)	1695 (1355–2984)	1713 ± 488.7	2198 ± 1305	2042 ± 727.1
**SOD**	373.2 (270.2–393.2)	421.3 (363.0–483.9)	347.8 (307.1–662.3)	394.6 ± 77.02	401.8 ± 129.7	473.4 ± 111.9
**CAT**	4618 ± 1819	4190 ± 2089	6449 ± 3693	6959 ± 1571	5380 ± 2094	5886 ± 2328
**GR**	20.49 (19.72–21.36)	15.42 (6.65–34.04)	22.36 (12.66–60.10)	22.16 (8.47–54.66)	21.85 (16.15–61.75)	33.44 (13.13–70.75)
**GPx**	111.3 ± 70.07	96.38 ± 40.59	133.9 ± 30.07	149.8 ± 34.86	112.2 ± 55.68	109.3 ± 19.58
**GST**	58.50 (33.03–68.24)	9.95 (4.21–75.26)	81.71 (59.36–95.33)	182.7 ± 80.27	152.6 ± 66.13	169.1 ± 56.58

^1^ Statistical analysis for non-parametric data was performed using the Kruskal–Wallis non-parametric test followed by Dunn’s pairwise multiple comparison test, and data is presented as the median and IQR (25th–75th quartile). For parametric data, ANOVA followed by Tuckey’s pairwise comparison test was used, with results presented as mean ± SD. ROS: µmol DCF/mg protein; MDA: µmol MDA/mg protein; PC: nmol NADH/min·mg protein; GSH: µmol GSH/mg protein; GSSG: µmol GSSG/mg protein; SOD: U/mg protein; CAT: U/mg protein; GR: nmol NADPH/min·mg protein; GPx: nmol NADPH/min·mg protein; GST: nmol CDNB/min·mg protein.

**Table 3:** Evaluation of oxidative stress parameters on the kidney after exposure to 5 (K5) or 10 (K10) mg/L of ketamine. The experimental groups have at least four samples (*n* = 4) each ^1^.

	Female			Male		
	Control	K5	K10	Control	K5	K10
**ROS**	11.71 ± 2.85	11.80 ± 4.99	12.03 ± 2.52	15.46 ± 5.39	17.33 ± 3.22	20.01 ± 4.82
**MDA**	2.27 (0.80–2.46)	2.06 (1.77–4.50)	2.06 (1.49–2.92)	2.25 ± 0.79	2.60 ± 1.25	2.63 ± 0.91
**PC**	6.38 ± 1.47	5.78 ± 2.41	4.05 ± 1.02	6.48 ± 4.39	7.95 ± 5.29	5.58 ± 1.82
**GSH**	3.16 (1.69–7.23)	3.39 (2.35–4.92)	3.61 (3.17–4.19)	4.39 ± 1.69	4.76 ± 2.14	3.72 ± 1.64
**GSSG**	26.76 ± 9.62	26.27 ± 10.52	28.27 ± 9.56	24.27 ± 7.02	31.53 ± 14.14	28.52 ± 11.64
**SOD**	171 (76.26–208.8)	150.3 (116.0–208.3)	178.2 (158.0–300.8)	337.07 ± 62.87	271.63 ± 96.79	242.8 ± 77.09
**CAT**	2319 ± 779.03	2574 ± 932.25	1794 ± 932.79	2047 ± 1094	3194 ± 935.3	2943 ± 1156
**GR**	29.10 ± 2.91	39.92 ± 27.67	43.0 ± 25.20	34.45 ± 42.74	36.97 ± 24.60	40.59 ± 32.17
**GPx**	81. 70 ± 38.33	104.17 ± 55.78	123.59 ± 86.93	75.20 ± 21.64	97.22 ± 14.52	66.15 ± 29.53
**GST**	64.02 ± 31.23	63.28 ± 36.22	79.81 ± 32.69	60. 58 ± 35.71	47.76 ± 17.07	55.89 ± 27.49

^1^ Statistical analysis for non-parametric data was performed using the Kruskal–Wallis non-parametric test followed by Dunn’s pairwise multiple comparison test, and data is presented as the median and IQR (25th–75th quartile). For parametric data, ANOVA followed by Tuckey’s pairwise comparison test was used, with results presented as mean ± SD. ROS: µmol DCF/mg protein; MDA: µmol MDA/mg protein; PC: nmol NADH/min·mg protein; GSH: µmol GSH/mg protein; GSSG: µmol GSSG/mg protein; SOD: U/mg protein; CAT: U/mg protein; GR: nmol NADPH/min·mg protein; GPx: nmol NADPH/min·mg protein; GST: nmol CDNB/min·mg protein.

**Table 4:** Evaluation of liver and kidney function after exposure to 5 (K5) or 10 (K10) mg/L of ketamine. The experimental groups have at least three samples (*n* = 3) each ^1^.

		Female			Male		
		Control	K5	K10	Control	K5	K10
**Liver Function**	**AST**	81.70 (58.30–105.0)	235.7 (105.8–246.2)	113.5 (48.80–265.3)	144.1 ± 64.36	115.3 ± 56.04	87.16 ± 60.76
	**ALT**	5.70 (5.20–6.10)	2.20 (1.73–3.28)	3.95 (2.60–6.18)	3.10 (2.60–3.50)	4.40 (3.50–7.0)	2.65 (1.80–3.50)
	**ALP**	57.80 (42.90–95.70)	116.4 (86.25–142.8)	86.60 (61.05–115.1)	38.0 (31.40–57.80)	50.35 (33.45–53.68)	53.65 (39.18–83.33)
**Kidney Function**	**Creatinine**	1.0 ± 0.13	1.64 ± 1.28	1.33 ± 1.04	1.07 (0.65–1.93)	0.80 (0.73–1.13)	0.47 (0.37–1.23)
	**BUN**	31.90 ± 9.93	25.28 ± 5.78	31.86 ± 10.45	24.13 (12.28–37.24)	31.21 (22.68–40.36)	25.80 (21.63–36.82)

^1^ Statistical analysis for non-parametric data was performed using the Kruskal–Wallis non-parametric test followed by Dunn’s pairwise multiple comparison test, and data is presented as the median and IQR (25th–75th quartile). For parametric data, ANOVA followed by Tuckey’s pairwise comparison test was used, with results presented as mean ± SD. AST: U/L; ALT: U/L; ALP: U/L; Creatinine: mg/dL; BUN: mg/dL.

**Table 5:** Evaluation of apoptosis, inflammation, and DNA damage parameters in the kidney after exposure to 5 (K5) or 10 (K10) mg/L of ketamine. The experimental groups have at least three samples (*n* = 3) each ^1^.

		Female			Male		
		Control	K5	K10	Control	K5	K10
**Apoptosis**	**Caspase 3**	75.82 (54.69–225.8)	68.96 (38.47–82.54)	69.12 (54.94–87.59)	77.22 ± 28.14	77.35 ± 26.49	72.32 ± 16.62
	**Caspase 9**	148.2 ± 98.12	99.94 ± 20.39	102.6 ± 19.45	114.2 ± 21.75	105.3 ± 19.59	111.4 ± 16.24
**Inflammation**	**NO**	0.82 (0.77–1.01)	0.57 (0.48–0.75)	0.75 (0.60–0.88)	0.71 (0.57–1.00)	0.93 (0.72–1.14)	0.71 (0.66–1.24)
**DNA damage**	**dsDNA breaks**	169.8 (92.81–217.0)	181.5 (142.2–243.2)	271.1 (130.5–460.5)	337.0 ± 146.3	191.7 ± 91.28	304.3 ± 304.9

^1^ Statistical analysis for non-parametric data was performed using Kruskal–Wallis non-parametric test followed by Dunn’s pairwise multiple comparison test, and data is presented as the median and IQR (25th–75th quartile). For parametric data, ANOVA followed by Tuckey’s pairwise comparison test was used, with results presented as mean ± SD. Caspase 3: µmol pNA/mg protein; Caspase 9: µmol pNA/mg protein; NO: µmol NO/mg protein; dsDNA breaks: µg dsDNA/mgProtein.

## Data Availability

All data supporting the findings described in this article will be provided by the authors upon request.

## Supplementary Materials

The following supporting information can be downloaded at: https://www.mdpi.com/article/10.3390/cimb47080592/s1.

## Abbreviations

The following abbreviations are used in this manuscript:

NMDA	N-methyl-D-aspartate
ROS	Reactive oxygen species
CI	Confidence interval
UTAD	University of Trás-os-Montes and Alto Douro
K5	5 mg/L of ketamine
K10	10 mg/L of ketamine
MDA	Malondialdehyde
PC	Protein carbonyls
GSH	Reduced glutathione
GSSG	Oxidized glutathione
SOD	Superoxide dismutase
CAT	Catalase
GR	Glutathione reductase
GPx	Glutathione peroxidase
GST	Glutathione S-transferase
ALT	Alanine aminotransferase
AST	Aspartate aminotransferase
ALP	Alkaline phosphatase
BUN	Serum urea nitrogen
ANOVA	Two-way analysis of variance
SD	Standard deviation
IQR	Interquartile range
