# Supplementary material for: A Preliminary Approach to Oral Low-Dose Ketamine Self-Administration in Mice (Mus musculus)
Source: Curr Issues Mol Biol. 2025 Jul 27;47(8):592. doi: 10.3390/cimb47080592 (PMC12384402; doi:10.3390/cimb47080592)
Supplement: Supplementary file 1 [file cimb-47-00592-s001.zip › cimb-3703600-supplementary.pdf]

Table S1. Female water intake (mL) per cage throughout the 16 days of the study. Data are separated by treatment group and its corresponding daily ketamine dose (mg/mL) is listed beneath.

		Water intake (mL)															
	**Cage**	**Day 1**	**Day 2**	**Day 3**	**Day 4**	**Day 5**	**Day 6**	**Day 7**	**Day 8**	**Day 9**	**Day 10**	**Day 11**	**Day 12**	**Day 13**	**Day 14**	**Day 15**	**Day 16**
**Control**	**5**	13.5	11	12	11.5	8.5	8.5	8.5	12	13.5	14	22	16	18.5	16	18.7	18.5
	**15**	16.5	12	11.5	14	13	13	13	13	12	13	21.5	16	18.5	14.5	16.7	20.5
**Dose (mg/mL)**		**0**	**0**	**0**	**0**	**0**	**0**	**0**	**0**	**0**	**0**	**0**	**0**	**0**	**0**	**0**	**0**
**K5**	**1**	14	11	13	12.5	13.3	13.3	13.3	12	12.5	13	23	22	19.5	14	17.5	15
	**2**	11	13.5	13.5	13.5	12.3	12.3	12.3	10.5	10.5	13	16.5	16.5	17	11.5	15.5	11
	**11**	12	8	12.5	11.5	11	11	11	9.5	11	14	14	13	15	12	14	19.5
	**12**	12	12.5	12.5	13.5	7.75	7.75	7.75	10	12	16	20.5	14	16	14.5	17.3	14
**Dose (mg/mL)**		**0.3**	**0.6**	**0.9**	**1.2**	**1.5**	**1.5**	**1.5**	**1.5**	**1.5**	**1.5**	**1.5**	**1.5**	**1.5**	**1.5**	**1.5**	**1.5**
**K10**	**3**	11	11	12.5	12	12.3	12.3	12.3	8	12.5	11.5	17	11.5	10	12	15.5	15
	**4**	12	8	11.5	13	14	14	14	11.5	11	12	14.5	24.5	13.5	11	14.2	14
	**13**	12	10	13	9.5	8.5	8.5	8.5	8	10.5	14	14	17	10	12.5	12.7	11.5
	**14**	12.5	6.5	10.5	10	10	10	10	7.5	8	13.5	18	17.5	11	13	13.0	14.5
**Dose (mg/mL)**		**0.6**	**1.2**	**1.8**	**2.4**	**3**	**3**	**3**	**3**	**3**	**3**	**3**	**3**	**3**	**3**	**3**	**3**

Table S2. Male water intake (mL) per cage throughout the 16 days of the study. Data are separated by treatment group and its corresponding daily ketamine dose (mg/mL) is listed beneath.

		**Water Intake (mL)**															
	**Cage**	**Day 1**	**Day 2**	**Day 3**	**Day 4**	**Day 5**	**Day 6**	**Day 7**	**Day 8**	**Day 9**	**Day 10**	**Day 11**	**Day 12**	**Day 13**	**Day 14**	**Day 15**	**Day 16**
**Control**	**10**	12.5	13.5	12	13	8.5	8.5	8.5	13	10	13.5	9	9	11	10	12.7	16.5
	**20**	14.5	14.5	14	13	9.7	9.7	9.7	12.5	11	14	14	14	13	12.5	14.2	12
**Dose (mg/mL)**		**0**	**0**	**0**	**0**	**0**	**0**	**0**	**0**	**0**	**0**	**0**	**0**	**0**	**0**	**0**	**0**
**K5**	**6**	12.5	9.5	12.5	10.5	7.75	7.75	7.75	8	12	12.5	20.5	11	11	10.5	15.0	22.5
	**7**	12.5	11.5	12	12	10.3	10.3	10.3	10	11.5	13	15	14	13	8.5	14.8	15
	**16**	12	11	13	12.5	9.7	9.7	9.7	12	12	12	16	13	13	17	15.0	14
	**17**	12	8	14.5	7.5	9.3	9.3	9.3	8	9	12	8	14	12.5	9	14.3	7
**Dose (mg/mL)**		**0.3**	**0.6**	**0.9**	**1.2**	**1.5**	**1.5**	**1.5**	**1.5**	**1.5**	**1.5**	**1.5**	**1.5**	**1.5**	**1.5**	**1.5**	**1.5**
**K10**	**8**	11.5	7	13	7.5	6.75	6.75	6.75	6	11	12.5	17.5	24	13.5	11	13.0	7.5
	**9**	8.5	4	12.5	9	13	13	13	5	12	13	15	16	8.5	11	10.2	8.5
	**19**	11	6	14	7	11.7	11.7	11.7	6.5	6	12.5	7	10	7	11.5	9.8	13
**Dose (mg/mL)**		**0.6**	**1.2**	**1.8**	**2.4**	**3**	**3**	**3**	**3**	**3**	**3**	**3**	**3**	**3**	**3**	**3**	**3**

Table S3. Female ketamine intake (mg/kg) throughout the 16 days of the study calculated using the daily concentration administered in the water (mg/mL), water intake (mL), and the average weight (kg) of mice in each cage. Data are separated by treatment groups.

		Ketamine Intake (mg/kg)															
	**Cage**	**Day 1**	**Day 2**	**Day 3**	**Day 4**	**Day 5**	**Day 6**	**Day 7**	**Day 8**	**Day 9**	**Day 10**	**Day 11**	**Day 12**	**Day 13**	**Day 14**	**Day 15**	**Day 16**
**K5**	**1**	154.27	121.21	143.25	137.74	146.56	146.56	146.56	132.23	137.74	143.25	253.44	242.42	214.88	154.27	192.84	165.29
	**2**	116.06	142.44	142.44	142.44	129.78	129.78	129.78	110.79	110.79	137.17	174.10	174.10	179.37	121.34	163.55	116.06
	**11**	144.26	96.17	150.27	138.25	132.24	132.24	132.24	114.21	132.24	168.30	168.30	156.28	180.32	144.26	168.30	234.42
	**12**	151.44	157.75	157.75	170.36	97.80	97.80	97.80	126.20	151.44	201.91	258.70	176.67	201.91	182.98	218.74	176.67
**K10**	**3**	238.55	238.55	271.08	260.23	266.74	266.74	266.74	173.49	271.08	249.39	368.66	249.39	216.86	260.23	336.13	325.29
	**4**	249.24	166.16	238.86	270.01	290.78	290.78	290.78	238.86	228.47	249.24	301.17	508.87	280.40	228.47	294.24	290.78
	**13**	296.39	246.99	321.09	234.64	209.94	209.94	209.94	197.59	259.34	345.79	345.79	419.88	246.99	308.74	312.85	284.04
	**14**	305.28	158.75	256.44	244.23	244.23	244.23	244.23	183.17	195.38	329.70	439.61	427.39	268.65	317.49	317.49	354.13

Table S4. Male ketamine intake (mg/kg) throughout the 16 days of the study calculated using the daily concentration administered in the water (mg/mL), water intake (mL), and the average weight (kg) of mice in each cage. Data are separated by treatment groups.

		Ketamine Intake (mg/kg)															
	**Cage**	**Day 1**	**Day 2**	**Day 3**	**Day 4**	**Day 5**	**Day 6**	**Day 7**	**Day 8**	**Day 9**	**Day 10**	**Day 11**	**Day 12**	**Day 13**	**Day 14**	**Day 15**	**Day 16**
**K5**	**6**	113.71	86.42	113.71	95.52	70.50	70.50	70.50	72.78	109.17	113.71	186.49	100.07	100.07	95.52	136.46	204.69
	**7**	121.94	112.19	117.06	117.06	100.48	100.48	100.48	97.55	112.19	126.82	146.33	136.57	126.82	82.92	144.70	146.33
	**16**	128.99	118.24	139.73	134.36	104.26	104.26	104.26	128.99	128.99	128.99	171.98	139.73	139.73	182.73	161.23	150.48
	**17**	127.99	85.33	154.65	79.99	99.19	99.19	99.19	85.33	95.99	127.99	85.33	149.32	133.32	95.99	152.88	74.66
**K10**	**8**	197.10	119.97	222.81	128.54	115.69	115.69	115.69	102.84	188.53	214.24	299.94	411.34	231.38	188.53	222.81	128.54
	**9**	152.83	71.92	224.75	161.82	233.74	233.74	233.74	89.90	215.76	233.74	269.70	287.68	152.83	197.78	182.80	152.83
	**19**	235.63	128.53	299.89	149.95	250.62	250.62	250.62	139.24	128.53	267.76	149.95	214.21	149.95	246.34	210.64	278.47
